# Narcolepsy, depression, and severe flushing in an obese man

**DOI:** 10.1002/ccr3.2873

**Published:** 2020-08-10

**Authors:** Fatimo Biobaku, Adel Hanna, George Matthews, Sandeep Dhindsa, Paresh Dandona

**Affiliations:** ^1^ Division of Endocrinology, Diabetes and Metabolism State University of New York at Buffalo Buffalo New York; ^2^ Division of Cardiology State University of New York at Buffalo Buffalo New York; ^3^ Division of Endocrinology, Diabetes and Metabolism Saint Louis University Saint Louis Missouri

**Keywords:** hot flushes, hypogonadism, narcolepsy, severe obesity

## Abstract

Hypogonadism as a cause of depression, daytime sleepiness, and flushing is often missed in young males. Our case report highlights the importance of screening for hypogonadotropic hypogonadism and its treatment in symptomatic men with severe obesity, especially if they have depression, excessive sleepiness, and narcolepsy.

## Graphical abstract

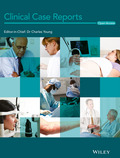

## 1 CASE REPORT

A 39‐year‐old man with episodic flushing was referred to our center by his cardiologist for evaluation for possible carcinoid syndrome or pheochromocytoma. The patient presented with a history of severe generalized cutaneous flushing which was more prominent on the face, with profuse sweating following every meal. He also reported intermittent bouts of diarrhea. These symptoms were not specific to a particular kind of food and had been ongoing for at least a year. There was no history of abdominal pain, weight loss, or bloody stools. He had a history of unexplained chest pain for which he was seen by a cardiologist. He also had lower extremity edema which was attributed to venous insufficiency, confirmed by a duplex scan. The patient had hypertension, and he was also on CPAP for sleep apnea. There was no difficulty in breathing, wheezing, or cough. He reported no palpitations or headache. In addition, he complained of a marked reduction in his energy level. On a scale of 1‐10, he reported an energy level of 2/10. The patient did not have diabetes.

Inquiries into the patient's sexual function revealed a significantly decreased libido with erectile dysfunction, loss of early morning erections, excessive sleepiness during the daytime, and depression for which he was seeing a psychiatrist. He had no history of testicular trauma, pain, or swelling. His CPAP treatment had been optimized, and sleep apnea did not account for his daytime sleepiness. His medications included aripiprazole (for depression) and adderall (amphetamine/dextroamphetamine) for sleepiness during the day which was diagnosed as narcolepsy. For his hypertension, he was being treated with amlodipine and lisinopril. The patient was also on diuretics, spironolactone, and furosemide for his edema. Prior to his presentation at our center, he was evaluated by two endocrinologists for possible carcinoid syndrome on account of his bothersome flushing, diarrhea, and accompanying symptoms. Urinary 5‐HIAA excretion was marginally elevated on both occasions (Table 1).

**Table 1:** Results of laboratory investigations

Prior to presentation		
Urine 5‐HIAA (mg/24 h)	Result	Reference range
1st evaluation	9.7	<6
2nd evaluation	7	<6
Evaluation at our center		
Plasma catecholamines (pg/mL)		
Dopamine	33	<30
Epinephrine	38	<50
Norepinephrine	753	112‐658
Serum serotonin (ng/mL)	25	56‐244
Thyroid function tests		
T4, total	6.3 mcg/dL	4.5‐12.0
T3 uptake	33.10%	22‐35
TSH	1.36 mIU/L	0.4‐4.5
Gonadotropins (mIU/mL)		
FSH	1.3	1.6‐8.0
LH	3.8	1.5‐9.3
Baseline Testosterone (ng/mL)		
Testosterone, total	181	250‐827
Testosterone at 3 mo (ng/dL)		
Testosterone, free	15.8	3.5‐15.5
Testosterone, total	698	
Testosterone at 6 mo after treatment (ng/dL)		
Testosterone, free	15.7	
Testosterone, total	775	

A colonoscopy carried out by a gastroenterologist for his diarrhea revealed benign polyps but no evidence of a tumor. Family history was significant for a history of myocardial infarction in his father. The patient was not a smoker and he was allergic to CT contrast dye. In addition to the above, he had been obese from his childhood and has been unsuccessful in losing weight after several attempts. The patient is married without children. Physical examination revealed severe obesity (weight—133.8 kg, BMI—43.6 kg/m^2^), a depressed mood, and blood pressure of 135/85 mm Hg. The patient's sense of smell was intact, there was no gynecomastia and no abnormalities in the external genitalia. His Epworth Sleepiness Scale (ESS) score was 20 out of a maximum of 24. His Swiss Narcolepsy Scale (SNS) was −34, consistent with narcolepsy and cataplexy. Both of these indices, ESS and SNS, were consistent with the diagnosis of narcolepsy.

His laboratory investigations revealed normal complete blood counts, including differential white blood and platelet counts, and normal plasma electrolytes, albumin, creatinine, and eGFR. Morning cortisol, IGF1, and prolactin concentrations were also normal. Serum alanine aminotransferase and alkaline phosphatase were mildly elevated. Plasma total testosterone concentration was low at 181 ng/dL while LH concentration was subnormal and FSH was low normal (Table 1). Plasma concentrations of thyroxine, thyroid‐stimulating hormone, and serotonin were normal (Table 1). The concentrations of dopamine, epinephrine and nor‐epinephrine were marginally elevated. This marginal increase was not consiedered clinically significant.

## 2 DIAGNOSIS AND PROGRESS

A diagnosis of obesity‐related hypogonadotropic hypogonadism was made on the basis of the history, low plasma testosterone, low LH, and low normal FSH. The flushing attacks, lack of energy, and depression were attributed to hypogonadism. We were not certain about the pathogenesis of excessive sleepiness and narcolepsy.

The patient consented to treatment with intramuscular testosterone following detailed counseling on potential benefits and harm including the effect on fertility. He was started on 200 mg/mL intramuscular testosterone injections every 2 weeks. At his 2‐month follow‐up visit, he reported an energy level of 9/10 with near disappearance of cutaneous flushing and sweating. The patient noted that mild episodes of cutaneous flushing occurred 1‐2 days preceding his next testosterone injection. His depression, narcolepsy, and episodic diarrhea had also resolved totally. His hematocrit was 50%, and the repeat total testosterone level was 698 ng/dL. Free testosterone was 15.8 ng/dL (Table 1). The patient then underwent bariatric surgery (sleeve gastrectomy).

The patient was reviewed again after 3 months, and he had lost 30 kg of weight. He reported a declining trend in his BP with associated lightheadedness, necessitating a discontinuation of amlodipine and his diuretic medications by his cardiologist. The patient's erectile dysfunction also improved, and he reported having morning erections. His hematocrit was 52.5%, and his repeat total testosterone level was 775 ng/dL, and free testosterone was 15.7 ng/dL (Table 1).

His testosterone dose was reduced to 150 mg every 2 weeks on account of his elevated hematocrit. Persistent erythrocytosis led to the cessation of testosterone treatment and the excessive sleepiness, depression, sluggishness, and lack of energy returned. He was advised to donate blood so as to reduce his hematocrit and restart testosterone treatment. Re‐institution of testosterone treatment led to the resolution of excessive sleepiness, narcolepsy, depression, and the lack of energy.

## 3 DISCUSSION

This patient clearly had hypogonadotropic hypogonadism associated with severe obesity which resulted in lack of libido, sexual performance, erectile dysfunction, generalized weakness, lack of energy, and frequent severe flushing. However, his most unique symptom was that of narcolepsy associated with depression, both of which, to our surprise, resolved following testosterone treatment, eliminating the need for amphetamine treatment. Narcolepsy has previously not been associated with clinical manifestations of male hypogonadism although a reduction in circulating LH levels has been documented in hypocretin‐deficient narcoleptic men despite normal gonadal hormone concentrations in plasma.^1^ Thus, the occurrence of narcolepsy and its resolution following testosterone makes this the first reported case of this kind. His narcolepsy previously had to be treated by the psychiatrist with an amphetamine preparation indicated for this condition. The fact that testosterone treatment led to the resolution of narcolepsy suggests that hypogonadism may have been causative in this case. This was further ascertained in this case since cessation of treatment due to erythrocytosis led to the recurrence of extreme sleepiness and narcolepsy. These symptoms resolved again after testosterone was restarted following blood donation and the reduction in hematocrit.

Although the signs and symptoms of testosterone deficiency in men are well described in the literature, it is easily missed and mistaken for other entities in the morbidly obese. Overt symptoms of testosterone deficiency such as hot flushes are more commonly seen in men with severe testosterone deficiency, as in patients on androgen deprivation therapy (ADT) for prostate cancer. Almost all (>80%) men with prostate cancer on androgen deprivation develop hot flashes once they have castrate testosterone concentrations.^2^ Hot flashes resolve after discontinuation of ADT in parallel with the recovery of serum testosterone concentrations.^2^ Although castrate testosterone concentrations are not necessary for hot flashes to develop, it is quite uncommon for young, obese, hypogonadal men to present with profound symptoms of hot flashes. Bariatric surgery resulted in weight loss and the resolution of hypertension such that he experienced postural hypotension. Antihypertensive therapy had to be stopped altogether. This is, however, not surprising since bariatric surgery results in a fall in vasoconstrictors, angiotensinogen, angiotensin II, renin and endothelin‐1, and an increase in vasodilators such as atrial natriuretic peptide.^3^

Our case report suggests that physicians should be aware of the strong association between obesity and hypogonadotropic hypogonadism in men who present with hot flashes.^4^ Twenty‐five percent of nondiabetic patients with obesity have hypogonadism,^5^ while one‐third of patients with type 2 diabetes have this condition.^6^ Most importantly, depression and narcolepsy can be features of male hypogonadism and should lead to an assessment of testosterone concentrations since it may be readily reversible following appropriate replacement. One clinical feature which remains somewhat a mystery in this case is the symptom of diarrhea which too resolved after testosterone therapy. It is, however, worthy of note that lower levels of LH have been described in men with irritable bowel syndrome (IBS), with a tendency for these men to have IBS symptoms negatively correlated with testosterone levels.^7^

## 4 CONCLUSION

This case highlights some of the multiple complications that occur in tandem with obesity, especially severe obesity. They include hypogonadotropic hypogonadism and hypertension. While testosterone deficiency in this patient was adequately treated by testosterone replacement with the resolution of symptoms of flushing, lack of energy and narcolepsy, bariatric surgery resulted in weight loss and the resolution of hypertension. Physicians should recognize the symptoms of testosterone deficiency which includes excessive sleepiness, depression and hot flushes, and appropriately screen for it in young, symptomatic obese males in order to prevent misdiagnosing this entity with other conditions.

## CONFLICT OF INTEREST

There is no conflict of interest.

## AUTHOR CONTRIBUTIONS

FB: wrote the first draft of the case report. AH, GM, and PD: retrieved and documented relevant information from the patient. SD and PD: revised the manuscript and provided useful insights.
